# Primary Aortic Sarcoma: Rare and with Heterogeneous Presentations

**DOI:** 10.1055/s-0041-1729914

**Published:** 2021-12-08

**Authors:** Angela Pucci, Andrea De Martino, Uberto Bortolotti

**Affiliations:** 1 Division of Pathology, University Hospital, Pisa, Italy; 2 Division of Cardiac Surgery, University Hospital, Pisa, Italy

In the December 2019 issue of *AORTA* , Tessitore et al 1 presented the case of intimal sarcoma of the descending aorta in a 48-year-old man, diagnosed by a combination of computed tomography, magnetic resonance imaging, and 18F–fluorodeoxyglucose positron emission tomography (PET). The definitive diagnosis was achieved with a left thoracoscopic surgical biopsy demonstrating a primitive sarcoma of the aortic intima.

**Fig. 1:**
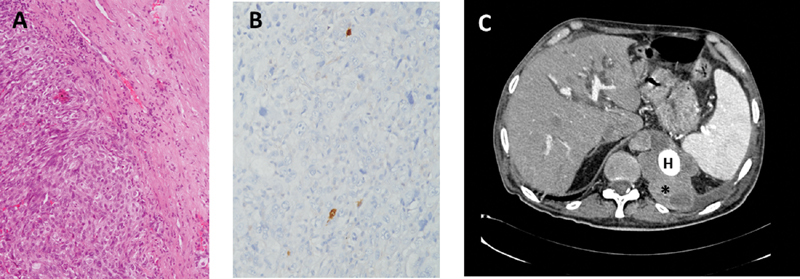
( **A** ) The aortic wall is infiltrated by high-grade and pleomorphic intimal sarcoma (hematoxylin and eosin, × 10), which shows, by immunohistochemistry, ( **B** ) nuclear expression of the amplified *MDM2* gene (immunoperoxidase technique with hematoxylin counterstaining, ×20). ( **C** ) Computed tomography, 1 year after surgery, shows neoplasm recurrence (asterisk *)* around the homograft (H) involving the esophagus and diaphragm.

Intimal sarcomas are extremely rare neoplasms, involving mostly the pulmonary artery and the aorta, with a dismal prognosis. 2 Since the clinical presentation is often nonspecific, preoperative diagnosis is difficult and most neoplasms are recognized only at histological analysis of surgical specimens. We reported a case similar to that described by Tessitore et al 1 in an elderly male patient, presenting with a pseudoaneurysm of the descending aorta, at the thoracoabdominal transition, close to the origin of the celiac trunk; the initial diagnosis of aortitis was justified by increased inflammatory blood markers, positivity of blood cultures, and intense tracer uptake in the aortic wall at 18F–fluorodeoxyglucose PET. 3 Pseudoaneurysm resection was performed through a left thoracotomy, combined with laparotomy with retroperitoneal approach, with the aid of left heart bypass, under moderate hypothermia, instituted by cannulating the left femoral artery, and the left inferior pulmonary vein. Suspecting the inflammatory nature of the aortic lesion, the pseudoaneurysm was transected and the aorta replaced with the interposition of a cryopreserved aortic homograft. Based on histology and immunohistochemistry, an intimal aortic sarcoma was diagnosed ( Fig. 1 ).

This case confirms the rarity and malignancy of neoplasms involving the aortic wall ( Fig. 1C ), the importance of routine histologic examination of the aortic wall specimens excised at surgery to allow the definitive diagnosis in patients with aortic pathologies 4 and the heterogeneity of clinical presentation that can mimic less serious aortic diseases. 3 For such reasons when reporting on uncommon diseases, such as those involving the aorta, previous important contributions on the same issue should not be overlooked.
